# Mental health profile of culturally and linguistically diverse (CALD) women entering New South Wales public prisons: a retrospective cohort study using real-world data

**DOI:** 10.1186/s40359-026-04401-z

**Published:** 2026-03-20

**Authors:** Reem Zeki, Alexandra Higgins, Scott Gill, Panayiota Zingirlis, Sharlene Kaye, Prabin Chemjong, Adam Vance, Mark V.A. Howard, Kimberlie Dean, Julia Bowman

**Affiliations:** 1 Research and Evaluation Team, Justice Health and Forensic Mental Health Network, Sydney, Australia; 2 School of Medicine and Public Health University of Newcastle, Newcastle, Australia; 3 Population and Preventative Health, Justice Health and Forensic Mental Health Network, Sydney, Australia; 4 Global Health and Equity Development Hub, La Trobe University, Melbourne, Australia; 5 Community Forensic Mental Health, Justice Health and Forensic Mental Health Network, Sydney, Australia; 6 Centre for Forensic Behavioural Science, Swinburne University of Technology, Melbourne, Australia; 7 National Drug and Alcohol Research Centre, University of New South Wales, Sydney, Australia; 8 Custodial Mental Health, Justice Health and Forensic Mental Health Network, Sydney, Australia; 9 Corrections Research Evaluation and Statistics, Corrective Services NSW, Sydney, Australia; 10 Discipline of Psychiatry and Mental Health, School of Clinical Medicine, University of New South Wales, Sydney, NSW Australia

**Keywords:** Australia, New South Wales, Culturally and linguistically diverse, CALD, Women, Mental health, Prison, Real-world data, Cohort study

## Abstract

**Background:**

Women in prison with culturally and linguistically diverse (CALD) backgrounds are at higher risk of marginalisation, isolation, and discrimination than non-CALD women. Despite this, the mental health of CALD women in prison is under-researched and there is limited evidence to inform practice. This study aimed to investigate the mental health profile of CALD women entering New South Wales (NSW) public prisons.

**Methods:**

A retrospective cohort study utilising routinely collected data managed by the Justice Health and Forensic Mental Health Network including all women entering NSW public prisons from 1 January 2015 to 30 June 2024. Reception Screening Assessment (RSA) forms were used to identify women who entered prison. Women were identified as CALD if they were born in countries other than the main English-speaking countries or their preferred language was not English. Mental disorders were identified from active health conditions recorded in the Justice Health electronic Health System. Descriptive statistics were produced for women’s characteristics and substance use and compared using Chi-square test. Multivariable logistic regression models were used to compare mental disorders between CALD and non-CALD women.

**Results:**

During the study period, 924 CALD and 3788 non-CALD women entered NSW public prisons. Reported substance use was lower in CALD than non-CALD women. CALD women were significantly less likely to have mental disorders than non-CALD women, except for schizophrenia spectrum and other psychotic disorders, which did not significantly differ in prevalence by CALD status. Depressive disorders were the most prevalent type of mental health disorder among CALD women (12.1%). The greatest difference was in bipolar-related disorders which were less prevalent among CALD than non-CALD women (1.4% vs. 4.5%, adjusted odds ratio (AOR) 0.35, 95% confidence interval (CI) 0.19–0.65).

**Conclusions:**

While CALD women had a lower recorded prevalence of mental disorders, this likely reflects a combination of systemic under-detection and differences in the characteristics and criminal justice pathways of CALD women in custody. A multifaceted approach is needed to ensure accurate identification and treatment, including culturally responsive practice, effective interpreter access, and creating an environment where women feel safe to report their mental health concerns.

**Supplementary Information:**

The online version contains supplementary material available at 10.1186/s40359-026-04401-z.

## Background

Australia is one of the most culturally diverse countries in the world, with more than a quarter (27.6%) of the population born overseas [1]. Australia’s migration patterns have transformed over time, resulting in increased cultural and linguistic diversity. In the first half of the 20th century, people born in English speaking countries were the main migrant group. However, recent migration statistics show a significant increase in the proportion of migrants from non-English speaking countries [1]. In 2021, there were approximately 5 million people living in Australia who were born outside of the main English speaking countries (MESC) (United Kingdom, Republic of Ireland, New Zealand, Canada, United States of America and South Africa [2]) and 5.8 million people reported using languages other than English at home, representing 19.6% and 22.8% of the general Australian population respectively [1, 3]. These statistics suggest that there are nearly 5 million people from culturally and linguistically diverse (CALD) backgrounds living in Australia representing 19.7% of the Australian general population.

Several indicators have been used to define CALD people, with the Australian Bureau of Statistics (ABS) utilising four core concepts to identify the CALD population. These concepts include country of birth, language spoken at home, English proficiency, and Aboriginal and/or Torres Strait Islander (hereafter, respectfully referred to as Aboriginal) identity [4]. In their 2021 systematic review, Pham et al. recommended defining CALD people as individuals who were born in non-English speaking countries or English not being their primary language, or both [5]. Additionally, to identify specific health needs and differences, Pham et al. suggested that Aboriginal people should be considered as a separate group [5].

CALD people are a heterogeneous group, with unique challenges related to resettlement and adjusting to a different society as well as varied pre- and post-migration experiences [6–8]. Additionally, CALD people have distinct social, religious, cultural and language differences [6, 7, 9]. These differences contribute to markedly diverse health profiles, patterns of service access, and health needs. Accordingly, any findings based on the CALD population as a single category should not be interpreted as uniform across cultural, linguistic, or migration subgroups; rather, they should be understood as reflecting aggregated data across a highly diverse population. Moreover, due to their pre-and post-migration experiences, CALD people, particularly refugees and asylum seekers, are at increased vulnerability to experiencing isolation and social disconnectedness [9]. Several factors may affect the physical and mental health of CALD people, as well as access to health services and their effective utilisation. These factors include language barriers, unemployment, social beliefs, stigma, a lack of information on available services coupled with low health literacy, as well as the limited availability of culturally informed health services [10–12].

Australia’s cultural and linguistic diversity underscores the importance of examining the characteristics and needs of CALD people within the prison system. Whilst Australia’s prison population is ethnically diverse, disparities persist in the representation of some minority groups. Prison data published by the ABS show that Aboriginal Australians and people born overseas represented 33% and 15% of the Australian prison population respectively [13]. In New South Wales (NSW), 20.3% of the prison population are people with CALD backgrounds [14].

Literature has identified that the healthcare needs of women in prison differ significantly to those of their male counterparts. Women in prison are more likely to report a history and current diagnosis of mental health illness, and are more likely to report having received treatment for a mental health problem [15, 16]. CALD women, especially those with limited English proficiency, are reported to be at higher risk of marginalisation, isolation and discrimination, have less knowledge of the available health services and subsequently report a poorer prison experience than non-CALD women [14, 17, 18]. In a study conducted by Western Sydney University on the experience of CALD women prior to, during and post imprisonment, women reported experiences of racism, social disconnection, and abuse [19]. There is also a high prevalence of trauma history among women in prison. For example, most women in prison not only have a history of sexual violence and early childhood abuse, but they are also at high risk of both physical and emotional abuse [20–22]. CALD women more specifically, are known to have experienced multiple traumas due to enforced traditional gender roles, domestic violence and discrimination [19, 23]. Similar findings have been observed in international research from high‑income countries with an over‑representation of minority ethnic groups such as the United Kingdom. Studies by Kelman and colleagues show that women in prison experience high rates of trauma, complex psychosocial disadvantage, and institutional environments that often fail to meet their needs [24, 25]. Additionally, women with trauma histories frequently perceive prison as retraumatising and report difficulties related to power imbalances and mistrust of authority [24, 25]. Kelman and colleagues (2024) also highlight the need for further research examining how women’s characteristics, including ethnicity, shape their experiences of imprisonment [24]. Similarly, Marzano et al. (2011) linked childhood trauma, discrimination, and adverse prison experiences to severe self‑harm among female prisoners [26].

Despite the negative implications the aforementioned factors have on mental health and wellbeing, there is limited available information on the mental health of CALD people in prison [27]. Two studies conducted by Rose et al. (2019, 2020) investigated mental health among CALD people in prison; however, these studies focused on the mental health of CALD men [28, 29]. To the authors’ knowledge, there are no studies investigating the mental health profile of CALD women in prison. To better understand the mental health profile of CALD women as well as their service needs, we conducted a study comparing the prevalence of mental disorders among CALD and non-CALD women entering NSW public prisons. We focused on six mental disorders: schizophrenia spectrum and other psychotic disorders, bipolar related disorders, depressive disorders, anxiety disorders, trauma and stress related disorders, and personality disorders.

## Methods

### Study design and data source

We conducted a retrospective cohort study utilising routinely collected administrative data managed by Justice Health and Forensic Mental Health Network (Justice Health NSW). We hypothesised that, among women in NSW prisons, the prevalence of mental disorders differs between CALD and non-CALD women.

Justice Health NSW is a state-wide specialty health network that provides health care to people (adults and young people) in contact with the NSW criminal justice and forensic mental health systems [30]. Justice Health NSW provides multidisciplinary health services to more than 30,000 people annually in several settings, including police cells, courts, correctional centres, youth justice centres, inpatient and community settings [30].

Datasets analysed for this research paper are listed below:

1. Reception Screening Assessment (RSA) data: RSA data is part of the Justice Health NSW electronic Health System (JHeHS). The RSA form is completed by Justice Health NSW registered or enrolled nurses upon entry to custody using a structured screening tool. It is used to conduct routine health assessments for all people within 24 h of entering custody, where possible. The form includes sections addressing physical, mental and population health, as well as recent substance use and a suicide/self-harm risk assessment. A women’s health section is also included for female entrants.
2. JHeHS active health condition data: JHeHS contains information on an individual’s current (active) physical and mental health conditions, gathered from three main sources:

i. Health conditions reported by people in prison at reception and subsequently confirmed by the community health provider.
ii. Health conditions reported at reception which have not been confirmed by a community health provider.
iii. Health conditions diagnosed by a Justice Health NSW health care provider during incarceration.

3. In JHeHS, health conditions may be recorded by a primary health nurse, a mental health nurse, or a specialised clinician. This multi‑provider pathway introduces the potential for measurement bias. For women from CALD backgrounds, interpreter access forms an integral component of the assessment process. When an interpreter is required but not immediately available, consultations are typically deferred and rescheduled with interpreter support, in accordance with Justice Health NSW policy, to ensure effective communication. Consequently, the timing of health condition recording may vary across individuals depending on interpreter availability and the point at which the assessment is completed. Women’s Health Assessment data: The Women’s Health Assessment form was available from March 2017 and completed by women’s health clinicians for women who have been referred for assessment and treatment for women’s health issues.

### Study population and duration

This study included all women, on remand and sentenced, aged 18 years or over who entered NSW public prisons from 1 January 2015 to 30 June 2024. The RSA date was used as a proxy for the date of prison entry as the majority of RSAs are done within 24 h of entering custody (72.4% overall; 75.0% among CALD women and 71.0% among non‑CALD women). As this study focused on women who entered NSW public prisons, RSAs completed in police cells or in court are not included in the study population.

### Study factors and outcome measurements

#### CALD women

For this study, country of birth and preferred language were used to identify CALD women. Women were classified as CALD if they were born in a non-MESC country, had a preferred language other than English, or both. To reduce the number of cases with “unstated” CALD status, self-identification at reception was used when information on both country of birth and preferred language was missing. The ABS list of MESC [2] was used to identify MESC and the women’s preferred language was used as a proxy for language spoken at home. Records of Aboriginal women were excluded from the analysis as this group has distinct health needs that differ from the general female CALD population [5, 31].

#### Mental disorders

The outcomes of the study include six mental disorders; each disorder consists of several mental health conditions. Mental disorders were recorded using a binary system where “1” indicates the presence of a specific mental disorder and “0” indicates its absence.

Mental health comorbidity was reported for women who had 2 or more mental disorders. The classification of these mental disorders is explained below.

The mental disorders are:

- Schizophrenia spectrum and other psychotic disorders
- Bipolar related disorders
- Depressive disorders
- Anxiety disorders
- Trauma and stress related disorders
- Personality disorders

Mental health conditions are recorded under active health conditions in JHeHS. Health conditions in JHeHS are classified based on Systemized Nomenclature of Medicine (SNOMED) classifications. For the purpose of this study, we grouped mental disorders based on the fifth edition of the Diagnostic and Statistical Manual of Mental Disorders (DSM-5) [32]. The DSM-5 classification lists the International Classification of Disease- Clinical Modification (ICD-CM) 9 and 10 codes against the mental health conditions. SNOMED codes were mapped according to the DSM and ICD criteria. The authors used clinical discretion to match unmapped codes to relevant diagnoses. All SNOMED-to-DSM‑5 mapping decisions were independently reviewed by the co‑authors, including psychologists, a psychiatrist, and mental health nurses. While some misclassification is possible in such mappings, it is expected to be non‑differential with respect to CALD status. Mental health conditions grouped under each mental health disorder are listed in the additional information (Additional Tables 1, 2, 3, 4, 5 and 6).

Data on the history of self-harm and suicide attempts are based on self-reported information at reception. Information on self-reported history of self-harm and suicide attempts was produced for women with an active mental health condition and those who did not have any of the examined active mental disorder.

#### Substance use

Data on substance use prior to prison entry are based on women’s self-report information at reception. The standard RSA process collects information on tobacco smoking up until prison entry and alcohol and drug use during the four weeks prior to entry. Current active alcohol and drug dependence, however, are recorded in the JHeHS health condition data. The drug dependence variable consists of any drug related dependence recorded in JHeHS health condition. Information on the type of drug dependence included in this variable is available in additional Table 7. Drug and alcohol dependence were recorded using a binary system where “1” indicates the presence of a specific mental disorder and “0” indicates its absence.

### Statistical analysis

Some women have multiple RSA records due to several episodes in custody. For the analysis, we used the most recent RSA record from the latest episode in custody within the study period. Active health conditions can be added to JHeHS during any imprisonment episode by Justice Health NSW health care providers. We included all mental health conditions that were active at the time the data was supplied. For this reason, the use of the last RSA episode was crucial as it is the closest episode to the time of the data supply. Numbers and proportions of women’s demographic characteristics (women’s age, number of episodes in public prisons, and number of children) and substance use were produced. Chi-square tests were used to compare women’s demographic characteristics and substance‑use patterns between CALD and non‑CALD groups, as these variables were categorical in nature.

Multivariable logistic regression was subsequently used to examine the association between CALD status (study factor) and each mental disorder (outcome factor), allowing us to adjust for potential confounders and estimate adjusted odds ratios (AORs). Crude and adjusted ORs and 95% confidence intervals (CI) were produced. Different models were utilised for each mental health disorder. The models included variables associated with the specific mental disorder in the univariable analysis (*P* < 0.2) in addition to factors identified in the literature as being associated with mental health. Variables tested in the univariable analysis included; age groups (years) (less than 20, 20–24, 25–29, 30–34, 35–39, 40–44, and 45 or more), if the women have children (yes, no), number of RSAs (groups) (first, 2–3, and 4 or more), reported smoking at reception (yes, no), drug use within 4 weeks prior to prison entry (yes, no), reported alcohol use frequency within four weeks prior to prison entry (no alcohol use, alcohol use daily or almost daily, alcohol use 2–3 times per week, and other alcohol use frequency), active alcohol dependence (yes, no), and active drug dependence (yes, no). During model development, we applied a backward stepwise selection procedure to identify and retain only the variables that remained statistically significant in the final model for that specific outcome. For this reason, the covariates included in each final model differ across disorders.

#### Dealing with missing records

Study factor: Records of women with missing CALD status were excluded from all analyses. We conducted a sensitivity analysis by re‑running the logistic regression models including women with ‘unknown’ CALD status as a separate category. The AORs and 95% confidence intervals from these updated models were very similar to those from the primary analysis, indicating that exclusion of women with missing CALD status did not materially influence the results.

Study outcomes: Mental disorders were recorded using a binary system, where ‘1’ indicates the presence of the disorder and ‘0’ indicates its absence. These variables did not include missing values.

Women’s demographic characteristic variables: The number of children was obtained from the Women’s Health Assessment data. Records where this information was unavailable either because the assessment occurred prior to March 2017 or because the form was not completed were coded as ‘not available’ and included in the analysis as a separate category. No other women’s demographic characteristic variables contained missing values. Substance use variables: Active alcohol dependence and active drug dependence were recorded as binary variables, with ‘1’ indicating use and ‘0’ indicating no use. These variables contained no missing values.

Variables with missing data were: Self-reported tobacco smoking at reception (missing 1.8%), alcohol use 4 weeks prior to prison entry (missing 0.3%), alcohol use frequency (missing 1.5%) and drug use 4 weeks prior to prison entry (missing 0.2%). For these variables, unknown or unrecorded values were coded as missing and handled accordingly during the analysis. For the logistic regression models, observations with missing values on any covariate were treated as missing for that specific variable. This approach ensured that only complete and valid data contributed to the estimation of covariate effects, while avoiding the introduction of bias that may occur from incorrectly assuming unknown values. No imputation was applied, as the intention was to preserve the integrity of the observed dataset.

The analysis was performed using SAS^®^ Enterprise Guide 8.3 software (SAS Institute, Inc) [33]. *P* values < 0.05 or CIs not including 1 were considered statistically significant.

### Ethics approval

This study is part of the People in NSW Public Prisons: Health Status and Service Utilisation project. Ethics approval for the project was granted from the Justice Health and Forensic Mental Health Network Human Research Ethics Committee (Reference number: 2020/ETH01927) and the Aboriginal Health and Medical Research Council Human Research Ethics Committee (Reference number: 1719/20).

## Results

8543 women entered NSW public prisons during the study period (9 years and 6 months) (from 1 January 2015 to 30 June 2024), 2989 (35.0%) of whom were Aboriginal and were therefore excluded from analysis. Of the remaining 5554 women comprising the study sample, 924 (16.6%) were CALD, 3788 (68.2%) non-CALD, and 842 (15.2%) were women whose CALD status was not stated in the data. There were 678 CALD women with known country of birth where 20.1% were born in Vietnam, 12.4% in China, 8.7% in Arabic speaking countries (Sudan, Lebanon, Iraq, Syria and Egypt), 6.2% in the Philippines and 4.7% in Thailand. Of the 443 CALD women with known preferred language, 62.8% of women reported a language other than English as their preferred language. Of these women, 33.5% reported Vietnamese, 10.8% reported Arabic and 9.0% reported Chinese as their preferred language.

Table 1 presents women’s demographic characteristics. The mean age for CALD women was 38.1 years (standard deviation (SD) 11.3) which was higher than the mean age for non-CALD women 35.6 years (SD 9.9). Although the proportion of women aged less than 25 years old was similar between CALD and non-CALD women (12.1% and 13.4%), the proportion of women aged 45 years or over was significantly higher among CALD compared to non-CALD women (28.0% vs. 19.1%, *p* < 0.001). A lower proportion of CALD women had been in prison multiple times (4 or more times) compared to non-CALD women (6.9% vs. 14.7%, *p* < 0.001). Of CALD women who had at least one Women’s Health Assessment, 85 (36.0%) women reported having 2 or more children. This proportion was significantly lower than the 45.2% reported by non-CALD women (*p* = 0.01) (Table 1).

**Table 1:** Women’s demographic characteristics by CALD status^a^

Women’s demographic characteristics	CALD (*n* = 924) *N* (%)	Non-CALD (*n* = 3788) *N* (%)	Total (*n* = 4712) *N* (%)	*P* value
Age groups (Years)				
Less than 20	13 (1.4)	88 (2.3)	101 (2.1)	< 0.001
20–24	99 (10.7)	421 (11.1)	520 (11.0)	
25–29	125 (13.5)	642 (16.9)	767 (16.3)	
30–34	140 (15.2)	690 (18.2)	830 (17.6)	
35–39	148 (16.0)	690 (18.2)	838 (17.8)	
40–44	140 (15.2)	533 (14.1)	673 (14.3)	
45 or more	259 (28.0)	724 (19.1)	983 (20.9)	
Number of RSAs completed in public prisons				
First	677 (73.3)	2064 (54.5)	2741 (58.2)	< 0.001
2–3	183 (19.8)	1167 (30.8)	1350 (28.7)	
4 or more	64 (6.9)	557 (14.7)	621 (13.2)	
Number of children^bc^				
None	97 (41.4)	393 (38.0)	490 (38.6)	0.04
One	54 (22.9)	174 (16.8)	228 (18.0)	
2–4	72 (30.5)	399 (38.6)	471 (37.1)	
5 or more	13 (5.5)	68 (6.6)	81 (6.4)	

*CALD* Culturally and linguistically diverse backgrounds

Notes: ^a^Excludes 842 (15.2%) women with unknown CALD status. ^b^For women who had a Women’s Health Assessment form completed. ^c^668 (74.5%) CALD women and 1034 (72.7%) non-CALD women did not have the Women’s Health Assessment form

The prevalence of substance use for CALD and non-CALD women is presented in Table 2. Generally, substance use was less prevalent among CALD women than non-CALD women. Less than half (43.1%) of CALD women reported smoking tobacco at reception, compared to 75.8% of non-CALD women (*p* < 0.001). Similarly, reported alcohol and drug use was lower among CALD than non-CALD women: (alcohol use: 16.2% vs. 25.5%, *p* < 0.001; drug use: 28.4% vs. 59.8%, *p* < 0.001) (Table 2).

**Table 2:** Substance use by CALD status^a^

Substance use	CALD *N* (%)	Non-CALD *N* (%)	Total *N* (&)	*P* value
Tobacco smoking at reception				
Yes	391 (43.1)	2821 (75.8)	3212 (69.4)	< 0.001
No	517 (56.0)	899 (23.7)	1416 (30.1)	
Not stated	16 (1.7)	68 (1.8)	84 (1.8)	
Alcohol use 4 weeks prior to prison entry				
Yes	150 (16.2)	965 (25.5)	1115 (23.7)	< 0.001
No	773 (83.7)	2812 (74.2)	3585 (76.1)	
Not stated	1 (0.1)	11 (0.3)	12 (0.3)	
Alcohol use frequency^b^				
Daily or almost daily use	45 (30.0)	420 (43.5)	465 (41.7)	0.02
2–3 times per week	18 (12.0)	104 (10.8)	122 (10.9)	
Other	85 (56.7)	426 (44.2)	511 (45.8)	
not stated use frequency	2 (1.3)	15 (1.6)	17 (1.5)	
Active alcohol dependence^c^	23 (2.5)	248 (6.6)	271 (5.8)	< 0.001
Drug use 4 weeks prior to prison entry				
Yes	262 (28.4)	2259 (59.8)	2521 (53.6)	< 0.001
No	660(71.4)	1520 (40.1)	2180 (46.3)	
Not stated	2 (0.2)	9 (0.24)	11 (0.2)	
Type of drug used^d^				
Benzodiazepines	38 (14.7)	415 (18.4)	453 (18.0)	0.309
Heroin	51 (19.6)	404 (17.9)	455 (18.1)	0.481
Prescribed opioids	6 (2.3)	84 (3.7)	90 (3.6)	0.326
Non prescribed opioids	< 5	88 (3.9)	np	0.103
Non prescribed pharmaceutical medications	0 (0.0)	24 (1.1)	24 (1.1)	**-**
Cannabis	66 (25.4)	903 (40.0)	969 (38.5)	< 0.001
New psychoactive substance	12 (4.6)	134(5.9)	146 (5.8)	0.648
Stimulants	165 (63.5)	1442 (63.9)	1607 (63.9)	0.936
Other	< 5	38 (1.7)	np	0.521
Top 5 active drug dependence^c^				
Opioid dependence	42 (4.6)	366 (9.7)	408 (8.7)	< 0.001
Benzodiazepines dependence	18 (2.0)	249 (6.6)	267 (5.7)	< 0.001
Amphetamine dependence	30 (3.3)	218 (5.8)	248 (5.3)	< 0.001
Cannabis dependence	8 (0.9)	193 (5.1)	201 (4.3)	< 0.001
Heroin dependence	21 (2.3)	171 (4.5)	192 (4.1)	0.002
Any active drug dependence^c^	87 (9.4)	823 (21.7)	910 (19.3)	< 0.001

*CALD* Culturally and linguistically diverse backgrounds, *np* not published

Notes: ^a^Excludes 842 (15.2%) women with unknown CALD status. ^b^For women who reported alcohol use 4 weeks prior to prison entry only. ^c^Active at the time of data supply. ^d^For women who reported drug use 4 weeks prior to prison entry only

In assessing the data on the type of substances used, the only significant difference was in the proportion of women using cannabis where 25.4% of CALD and 40.0% of non-CALD women reported using cannabis within 4 weeks prior to prison entry (*p* < 0.001) (Table 2).

Although there was no difference in the proportion of CALD and non-CALD women who reported using stimulants, the type of stimulants used varied between these two groups. Methamphetamine or amphetamines, or both, were the most commonly used type of stimulants in the 4 weeks preceding prison entry, reported by the overwhelming majority of both CALD (89.1%) and non-CALD (95.5%) women, although more commonly reported by non-CALD women (*p* < 0.001). A higher proportion of CALD women reported using cocaine compared to non-CALD women (11.5% vs. 3.7%, *p* < 0.001) (Table 2).

Analogous to the reported alcohol and drug use, active alcohol and/or drug dependence were less prevalent among CALD compared to non-CALD women (alcohol dependence: 2.5% vs. 6.6%, *p* < 0.001; drug dependence: 9.4% vs. 21.7%, *p* < 0.001) (Table 2). Of women with active opioid dependence, 18 CALD women (42.9%) and 168 non-CALD women (46.3%) (*p* = 0.673) reported being on Opioid Agonist Treatment (OAT) in the community at reception.

The prevalences of mental disorders are presented in Table 3. These estimates represent the active mental health conditions recorded in the JHeHS at reception or during custody. This includes conditions reported at reception and confirmed by community health providers, conditions reported but not yet confirmed, and conditions diagnosed by Justice Health NSW clinicians during incarceration. These figures therefore reflect recorded active diagnoses rather than lifetime or interview‑based prevalence.

**Table 3:** Mental disorders among CALD women compared to non-CALD women ^a^

Mental disorder	CALD *N* (%)	Non-CALD^b^ *N* (%)	OR (95% CI)	AOR (95% CI)
Schizophrenia spectrum and other psychotic disorders ^c^	33 (3.6)	172 (4.5)	0.78 (0.53–1.14)	0.79 (0.53–1.18)
Bipolar related disorders^d^	13 (1.4)	170 (4.5)	0.30*(0.17–0.54)	0.35*(0.19–0.65)
Depressive disorders^e^	112 (12.1)	769 (20.3)	0.54*(0.44–0.67)	0.62*(0.50–0.77)
Anxiety disorders^f^	35 (3.8)	414 (10.9)	0.32*(0.23–0.46)	0.37*(0.25–0.53)
Trauma and Stress related disorders^g^	18 (1.9)	191 (5.0)	0.37*(0.23–0.61)	0.47*(0.28–0.77)
Personality disorders^h^	16 (1.7)	175 (4.6)	0.36*(0.22–0.61)	0.47*(0.27–0.80)
Mental health comorbidity^i^	38 (4.1)	484 (12.8)	0.29*(0.21–0.41)	0.35*(0.24–0.49)

*CALD* Culturally and linguistically diverse backgrounds, *OR* Odds ratio, *AOR* Adjusted odds ratio, *CI* Confidence interval

Notes: ^a^Excludes 842 (15.2%) women with unknown CALD status. ^b^Reference group. ^c^Adjusted for age groups, have children, number of RSAs (groups), reported drug use. ^d^Adjusted for age groups, number of RSAs (groups), reported smoking at reception, reported drug use, and active drug dependence. ^e^Adjusted for age groups, have children, number of RSAs (groups), reported drug use, reported alcohol use frequency, active alcohol, and drug dependence. ^f^Adjusted for age groups, number of RSAs (groups), reported smoking at reception, reported drug use, reported alcohol use frequency, and drug dependence. ^g^Adjusted for number of RSAs (groups) and active alcohol dependence, reported drug use, reported alcohol use frequency, and drug dependence. ^h^Adjusted for age groups, have children, number of RSAs (groups), active alcohol dependence, reported drug use, and active drug dependence. ^i^For women who had 2 or more mental disorders. Adjusted for age groups, number of RSAs (groups), reported smoking at reception, reported drug use, reported alcohol use frequency, and active alcohol and drug dependence

* Significant *P* < 0.05

Table 3 also presents the results from the univariable and multivariable analysis. With the exception of schizophrenia spectrum and other psychotic disorders, the prevalence of all other mental disorders was significantly lower among CALD compared to non-CALD women. Compared to non-CALD, CALD women were 63% less likely to have anxiety (AOR 0.37, 95% CI 0.25–0.53), 65% less likely to have bipolar related disorders (AOR 0.35, 95% CI 0.19–0.65), 53% less likely to have trauma and stress related disorders (AOR 0.47, 95% CI 0.28–0.77), 53% less likely to have personality disorders (AOR 0.47, 95% CI 0.27–0.80), and 38% less likely to have depressive disorders (AOR 0.62, 95% CI 0.50–0.77). There was no statistically significant difference in the prevalence of schizophrenia spectrum and other psychotic disorders between CALD and non-CALD women (AOR 0.79, 95% CI 0.53–1.18) (Table 3).

Of women who had an active mental disorder, 30 CALD women (16.8%) and 239 non-CALD women (19.6%) (*p* = 0.370) reported previous suicide attempts whereas 37 CALD women (20.9%) and 345 non-CALD women (28.2%) (*p* = 0.042) reported previous self-harm. Among women who did not have any of the examined active mental disorders, 61 CALD women (8.3%) and 434 non-CALD women (17.3%) reported previous suicide attempts (*p* < 0.001), and 76 CALD women (10.4%) and 553 non-CALD women (22.1%) reported previous self-harm (*p* < 0.001).

## Discussion

### Key findings

This study of over 4,500 women entering prison across a 9.5-year period is the first to investigate the mental health profile of culturally and linguistically diverse women. Our results show that in comparison to non-CALD women, CALD women appeared to have a lower prevalence of mental disorders and of substance use. The only exception was in the cases of schizophrenia spectrum and other psychotic disorders where there was no statistically significant difference in prevalence according to CALD status.

The results produced by this study are inconsistent with previous quantitative research findings on the mental health of the Australian CALD population, noting that these previous studies have been conducted on CALD men in prison. Two studies were conducted by Rose et al. (2019, 2020) in a male maximum-security prison in Victoria [28, 29]. The 2019 study aimed to identify the prevalence of mental health factors including wellbeing, distress and coping whilst the 2020 study aimed to assess levels of psychological distress and mental illness symptoms [28, 29]. Both studies investigated mental health among three cross-cultural groups, people with CALD, Aboriginal and English-speaking backgrounds, and concluded that there was no significant difference in the psychological distress and mental health profile among cross-cultural groups [28, 29]. Similarly, our results are inconsistent with previous studies that were based in the community including both men and women. A study conducted by Shepherd et al. (2021) investigating the mental health of young people who attended mental health services in the community reported that there was no significant difference in mental health symptoms between young CALD and non-CALD people [34].

The inconsistencies between our study’s results and those preceding ours may be explained by several differing factors. Firstly, the study populations varied significantly between our study and those conducted previously. Both Rose et al. studies utilised a male population. It is well known that the mental health needs of women, both in prison and the general population, differ substantially from those of their male counterparts [28, 29, 35]. Secondly, the setting in which the preceding studies were conducted differed from the settings of our study. Both Rose et al. studies were conducted within a maximum-security prison, whilst our study population included all women who entered NSW public prisons which includes minimum, medium and maximum-security prisons. This difference may have important implications as the nature of the prison environment can affect the mental health of the people within them [36]. The Shepherd et al. study was conducted in a community setting which, due to the great variance of environmental and social factors, cannot be compared to the experience of those within prison [37, 38]. Lastly, our study methodology is different from that of the Rose et al. studies. Our study utilised routinely collected data of all women entering NSW public prison, while the Rose et al. collected data through cross sectional surveys of people who were post-reception and had been in prison for varying lengths of time. This difference in methodology (i.e., the time point at which data is collected) may also contribute to the difference in the prevalence of the mental health conditions found in the current study [16].

In our study, the lower proportions of mental disorders among CALD women compared to non-CALD women can be partially explained by two main factors. These are: (1) under diagnosis/recording of mental health conditions among CALD women and (2) protective factors common to CALD groups that are associated with a reduced risk of mental health problems.

### Barriers to accessing mental health services resulting in under diagnosis of mental disorders

CALD women often face intersecting challenges, including language barriers, stigma, culturally influenced help-seeking patterns, unequal access to services and broader systemic or structural inequities. Several barriers to the access of mental health services specific to CALD women have also been identified in the literature. Mehta et al. (2023) identified stigma as the biggest challenge to the access of mental health services [19]. In many CALD communities there is a great deal of stigma attached to mental disorders as well as access to services designed to provide care in this space. The stigma associated with mental health issues prevents people, particularly women, from accessing services for fear of social segregation and discrimination [35, 39–42]. The fear of community perceptions and stigma may lead CALD women or their families to minimise or mask their symptoms. These experiences can contribute to lower reporting rates of mental health and substance use, particularly in custodial settings. However, severe mental illness may be less sensitive to detection biases. In instances where women are affected by conditions such as schizophrenia, the chaotic and disruptive nature of a psychotic illness renders it difficult to avoid coming to the attention of emergency or mental health services, and/or the justice system and is less dependent on self-report [43, 44]. Thus, severe mental illness may be more likely to be diagnosed and treated than less severe mental illness. This may explain the non-significant difference in the rates of schizophrenia between CALD and non-CALD women. In addition to its effect on the access of mental health services, stigma is a major challenge in the uptake of drug and alcohol services [19]. Due to the social constructs and religious beliefs of many CALD communities, there is often greater shame, stigma and negative consequences attached to women reporting substance use [45]. These factors may, at least in part, explain the lower rate of reported substance use among CALD women.

Language is another key barrier to accessing mental health services. Australian prison systems are largely structured around English language communication which inherently places CALD people at a disadvantage when accessing healthcare [18]. The language barrier can affect multiple aspects of daily life prison including the ability to communicate their needs, understand instructions and routines, and meaningfully participate in prison activities and programs [14, 18, 19]. These can contribute to experiences of marginalisation with some women perceiving racism or discrimination due to this language barrier. Such experiences may further reduce their willingness to seek support or disclose psychological distress thus limiting their engagement with health services [17].

Unlike healthcare in the community, the restrictive nature of the prison environment creates additional barriers to accessing translation services. In these controlled and secure settings, both people in prison and healthcare providers have limited opportunities to access each other [46]. Additionally, the under-resourced system and high needs of the population [46] create competing priorities, limiting the opportunity to access quiet spaces and necessary devices for translation services. As a result, CALD women are often forced to rely on other inmates who can communicate in both English and their shared language for translation and to access prison services [17–19]. This reliance on other bilingual women within the prison setting has served to provide moral support and facilitate the services’ improved understanding of women’s needs [17]. However, peer translation has disadvantages such as lack of privacy, pressure on peers and the possibility of legal implications [17, 19]. In this study, English was not the preferred language for 278 CALD women, which indicates that language barriers could have affected up to 62.8% of all CALD women who entered NSW public prisons between 2015 and 2024.

CALD people, especially those from a refugee background, often have limited access to information on available health and social services [7, 47–50]. Similarly, in prison, one of the obstacles to engagement with mental health services is the lack of information on the availability of these services [19]. This specifically affects the CALD population as health information, such as brochures and information on prison-based tablets, may not be provided in all community languages, which creates additional barriers to accessing this information [19]. The language barrier and the lack of information on the availability of mental health services in prison have a compounding effect on CALD women’s access to health services. In addition to the lack of information on available services, access to programs and services in prison may be affected by institutional factors. In prison, programs and services such as wellbeing services and programs designed to help people to cope with and respond to stressful situations, are a lower priority compared to time-sensitive and criminogenic programs, as corrective services agencies have to prioritise providing programs to sentenced women before women on remand [51]. Additionally, the high mobility between centres for women on remand can be a barrier preventing women from accessing services or completing programs [51]. Whilst this applies to both CALD and non-CALD women, CALD women are more likely to experience a lengthier remand period due to limited access to legal services and therefore remain longer without the provision of mental health services [19]. Literature on access to health services by CALD groups also identified other factors that affect access to services including lack of services, specifically culturally competence services, long waiting time, mistrust, and racism [9, 18, 19, 29, 47–50].

Taken together, these findings suggest that multiple mechanisms may contribute to the lower recorded prevalence of mental disorders among CALD women. Systemic and structural factors, including under-detection, language and communication barriers, stigma affecting disclosure, and institutional constraints on assessment and documentation, are likely to play an important role in shaping recorded prevalence and may obscure underlying morbidity. At the same time, differences in the characteristics of CALD women in custody relative to non-CALD women, arising from composition or selection effects and differing pathways into the criminal justice system, may also contribute to observed prevalence patterns. In addition, potential protective influences within some CALD communities, as discussed below, may be relevant in specific contexts. Together, these explanations highlight the heterogeneity of CALD populations and suggest that the relative importance of these mechanisms may vary across subgroups and circumstances, rather than reflecting a single dominant explanation.

### Protective factors may contribute to the lower prevalence of mental disorders

Previous literature shows that the CALD population in prison have fewer and less complex criminogenic needs than the non-CALD population. For instance, Howard and Lobo’s (2020) study indicated that CALD people in NSW prisons were assessed as having a lower risk of reoffending than non-CALD people and were less likely to be in prison due to violent offences [14]. Additionally, Howard and Lobo found that CALD people in prison were less likely to have alcohol and other drug related criminogenic needs than non-CALD people [14]. Consistent with this finding, CALD women in our study reported lower rates of substance use than non-CALD women. Further, we found that CALD women have a lower prevalence of active drug dependence than non-CALD women. While we acknowledge that language barriers, stigma, and culturally shaped help-seeking behaviours can influence detection, these factors are unlikely to fully account for the systematically lower prevalence of conditions such as substance dependence, which typically present with clinically evident signs during reception screening. Instead, differences in pre-incarceration exposures and protective or risk factors such as cultural norms regarding substance use, migration selection effects, family and community structures, and differential socioeconomic pathways plausibly shape CALD women’s criminogenic and mental health profiles.

Taken together, these observations, alongside prior literature, suggest that this difference in CALD women’s criminogenic profile may reflect variance in a range of psychosocial factors hypothesised in the literature to be associated with, or contributing to, mental health outcomes.

CALD women in prison originate from various countries of birth and may have been separated from some or all their family and community networks. However, prior research indicates that they may be able to find solidarity with other inmates through their shared lived experiences in a prison system which is predominantly designed for males who can speak English [18, 19]. Being born overseas in non-English speaking countries can assist in forming a unique identity for CALD women in prison, providing a safe space for women to share their experience of exclusion within prison [19]. It also provides an opportunity for peer-support through assisting each other in translation [17, 19]. Some programs in prison have built upon this peer translation practice to create a formal peer support group for CALD women aimed at helping women navigate the system, provide cultural and linguistic support and ongoing relationships and safety [17].

Previous literature also indicates that religious beliefs may serve as a protective factor for mental health amongst CALD populations [42, 52, 53]. This is particularly seen amongst those from refugee backgrounds where religious beliefs may play a vital role and contribute to the difference in mental health profiles between CALD and non-CALD populations [17, 42, 52, 53]. Previous literature suggests that religious beliefs and practices are used as a coping mechanism when dealing with prison stress [28]. People in prison who hold religious beliefs have been found to have less depressive symptoms and a better prognosis for other mental health problems such as bipolar disorder and posttraumatic stress disorder [52]. Religious belief is also found to be associated with a reduction in suicide behaviour [54, 55]. Religious beliefs and practices may partially explain the lower rate of self-harm and suicide attempts reported by CALD women in our study, although the difference in suicide attempts was not statistically significant. We note that the references to protective factors are advanced as literature‑based hypotheses to contextualise our findings, rather than as explanations supported by the study’s primary data.

### Strengths and limitations

Our study is the first study to investigate the mental health profile and needs of CALD women entering NSW public prisons. The use of routinely collected data with a large sample size over an extended period of time provides the opportunity to investigate the health of population subgroups such as the CALD population and maximise the study power to identify marginal differences in health outcomes [56]. The use of routinely collected data also reduces the risk of information biases inherent in studies that rely on retrospective recall. Additionally, the inclusion of all CALD and non-CALD women entering prison reduces the risk of selection biases and provides population evidence that could be generalised to all Australian prison populations.

As this study relies on recorded mental health conditions captured in prison administrative data, there may be an underestimate of the true prevalence of mental disorders across the cohort. This is of increased relevance when specifically interpreting the results for CALD women, where factors such as language barriers and stigma may contribute to even lower rates of reporting. Another limitation of the administrative data is the missing data on country of birth and preferred language, which resulted in a high proportion of participants with unknown CALD status (18.8%). To reduce the proportion of missing data, we utilised self-reported CALD status at reception if both country of birth and preferred language data was unknown. By doing this we were able to reduce the proportion of unknown CALD status to 15.2%. We also conducted a sensitivity analysis by re-running the logistic regression models including women with ‘unknown’ CALD status as a separate category. The AORs and 95% confidence intervals from these updated models were very similar to those from the primary analysis, indicating that exclusion of women with missing CALD status did not materially influence the results.

The number of children indication, derived from the Women’s Health Assessment form, was only available for 1,398 women. This form was only available from March 2017 and was completed for 25.2% of our study population. The diagnostic information in this study was extracted from participants medical records and not from in-study structured interviews. Furthermore, the Justice Health NSW data lacked information on some socio-demographic and criminogenic factors that may differ between CALD and non-CALD women. These factors include domestic violence, education, housing, length of sentence, and index offence. The absence of these variables may introduce bias into the estimated results. Data linkage studies can be considered in future studies to address this limitation. Additionally, people at reception may have been reluctant to divulge mental health and substance misuse issues to Justice Health NSW staff as they are not independent research staff which may result in an under reporting of mental health problems and substance use. An additional limitation relates to the timing of the RSA. Although the dataset indicates whether the RSA occurred within 24 h of admission, the exact admission date was not available, preventing calculation of the precise interval between admission and RSA completion.

## Conclusion and recommendations

CALD women in prison have a significantly lower prevalence of mental health conditions than non-CALD women, with the exception of schizophrenia spectrum and other psychotic disorders. However, this lower prevalence may reflect under‑reporting or under‑recording among CALD women and may also reflect the under-identification of CALD status among women entering custody and should not be interpreted as indicating lower need or reduced vulnerability. Rather, the findings highlight the importance of distinguishing between documented prevalence and potential unmet mental health needs, emphasising an equity‑focused approach to ensuring appropriate assessment, support and care.

Previous literature suggests that this may be accounted for by lower mental health service utilisation by CALD populations, contributing to an underdiagnosis of mental health conditions. This may be due to several factors such as the fear of stigma, the experienced language barriers and lack of culturally informed information and services. These factors may partially explain the lower prevalence of mental health conditions among CALD women in prison.

On the other hand, CALD women in prison may draw on several protective factors that contribute to their resilience and ability to cope with mental health challenges. These factors include strong cultural identity, social support systems and religious or spiritual practices.

This study further highlights that more research is required to investigate mental health conditions amongst CALD populations in prison, how they utilise mental health services in prison, their experience of accessing these services and the presence of protective factors. Further research using other methods such as linked data, which draw on multiple sources of CALD status identification and diagnostic information, and incorporating culturally appropriate screening tools can help improve the reporting and identification of mental disorders. Research designed to ascertain whether CALD women living with psychosis are at a higher risk of contact with the criminal justice system is also recommended.

While some overarching mental health challenges faced by CALD populations may be similar across countries, these findings cannot be generalised beyond the Australian context. Each country has CALD communities with different cultural backgrounds, migration histories, and help seeking norms, resulting in unique mental health needs and barriers. As such, dedicated research is required in each setting, replicating the methods in this study and/or utilising other recommended methods, where appropriate, to explore mental health needs in diverse international contexts.

Addressing the mental health needs of CALD women in prison requires a multifaceted approach to ensure the accurate identification of CALD status and mental health conditions and treatment of mental health needs. This includes improving cultural competence amongst healthcare providers, ensuring access to interpreters at reception and throughout the period of incarceration, using culturally appropriate screening tools, and creating an environment where women feel safe to report their mental health concerns. By prioritising these measures, prison systems can increasingly support the mental health and overall wellbeing of CALD women, ultimately contributing to more effective rehabilitation and successful reintegration into the community. Failure to address these issues can perpetuate a cycle of neglect and marginalisation thus undermining the rehabilitation aims of the correctional system.

## Supplementary Information

Supplementary Material 1: Additional table 1. Mental health conditions grouped under schizophrenia spectrum and other psychotic Disorders. Additional table 2. Mental health conditions grouped under bipolar and related disorders. Additional table 3. Mental health conditions grouped under depressive Disorders. Additional table 4. Mental health conditions grouped under anxiety disorders. Additional table 5. Mental health conditions grouped under trauma and Stress related disorders. Additional table 6. Mental health conditions grouped under personality disorders. Additional table 7. Type of drug related dependence included in the drug dependence variable.

## Data Availability

Datasets utilised for the analysis of this study is not for sharing as ethical approvals for this project require that the data used in this analysis not be shared to protect privacy and confidentiality.

## Abbreviations

ABS: Australian Bureau of Statistics
AOR: Adjusted Odds Ratio
CALD: Culturally and linguistically diverse
CI: Confidence interval
DSM: Diagnostic and Statistical Manual
JHeHS: Justice Health electronic Health System
Justice Health NSW: Justice Health and Forensic Mental Health Network
ICD-CM: International Classification of Disease- Clinical Modification
MESC: Main English-speaking countries
NSW: New South Wales
OAT: Opioid Agonist Treatment
OR: Odds Ratio
RSA: Reception Screening Assessment
SNOMED: Systemized Nomenclature of Medicine
