# Supplementary material for: Mental health profile of culturally and linguistically diverse (CALD) women entering New South Wales public prisons: a retrospective cohort study using real-world data
Source: BMC Psychol. 2026 Mar 20;14:626. doi: 10.1186/s40359-026-04401-z (PMC13126866; doi:10.1186/s40359-026-04401-z)
Supplement: Supplementary file 1 — Supplementary Material 1: Additional table 1. Mental health conditions grouped under schizophrenia spectrum and other psychotic Disorders. Additional table 2. Mental health conditions grouped under bipolar and related disorders. Additional table 3. Mental health conditions grouped under depressive Disorders. Additional table 4. Mental health conditions grouped under anxiety disorders. Additional table 5. Mental health conditions grouped under trauma and Stress related disorders. Additional table 6. Mental health conditions grouped under personality disorders. Additional table 7. Type of drug related dependence included in the drug dependence variable. [file 40359_2026_4401_MOESM1_ESM.docx]

**Additional table 1: Mental health conditions grouped under schizophrenia spectrum and other psychotic Disorders**

| **Mental health condition** | **SNOMED CODE** | **ICD-9-cm**  **(ICD-10-cm) in DSM-5** |
| --- | --- | --- |
| Schizophrenia | 58214004 | 295.90 (F20.9) |
| Schizotypal personality disorder | 31027006 | 301.22 (F21) |
| Schizophrenia, unspecified | 192327003 | 298.9 (F29) |
| Schizophrenic disorders | 191526005 | Not in DSM-5 |
| Schizophrenic prodrome | 247804008 | Not in DSM-5 |
| Delusional disorder | 48500005 | 297.1 (F22) |
| Schizophreniform disorder | 88975006 | 295.40 (F20.81) |
| Schizoaffective disorder | 68890003 | No ICD code |
| Schizoaffective disorder, depressive type | 84760002 | 295.70 (F25.1) |
| Schizoaffective disorder, manic type | 271428004 | Not in DSM-5 |
| Schizoaffective disorder, mixed type | 270901009 | Not in DSM-5 |
| Organic catatonic disorder | 231442003 | 293.89 (F06.1) |
| Other nonorganic psychotic disorders | 192346002 | Not in DSM-5 |
| Unspecified nonorganic psychosis | 268698005 | Not in DSM-5 |
| Other schizophrenia | 268691004 | 298.8 (F28) |
| Other schizoaffective disorders | 192344004 | Not in DSM-5 |
| Simple schizophrenia | 191527001 | Not in DSM-5 |
| Acute transient psychotic disorder | 231489001 | Not in DSM-5 |

**Additional table 2: Mental health conditions grouped under bipolar and related disorders**

| **Mental health condition** | **SNOMED CODE** | **ICD-9-cm**  **(ICD-10-cm) in DSM-5** |
| --- | --- | --- |
| Bipolar affective disorder, current episode manic | 191618007 | Not in DSM-5 |
| Bipolar affective disorder, current episode mixed | 192362008 | Not in DSM-5 |
| Bipolar affective disorder, currently depressed, severe, with psychosis | 191632009 | 296.54 (F31.5) |
| Bipolar affective disorder, currently manic, severe, with psychosis | 191623007 | 296.44 (F31.2) |
| Bipolar affective disorder, unspecified | 192365005 | Not in DSM-5 |
| Bipolar disorder | 13746004 | Not in DSM-5 |
| Bipolar disorder in remission | 85248005 | 296.55 (F31.75) or 296.56 (F31.76) |
| Bipolar Disorder with mild to moderate depression | 66631006 | Not in DSM-5 |
| Bipolar I disorder, most recent episode hypomanic | 31446002 | Not in DSM-5 |
| Manic bipolar I disorder | 68569003 | Not in DSM-5 |
| Other bipolar affective disorders | 268701002 | 296.80 (F31.9) |
| Severe depressed bipolar I disorder without psychotic features | 61403008 | 296.53 (F31.4) |
| Cyclothymia | 76105009 | - 1. (F34.0) |

**Additional table 3: Mental health conditions grouped under depressive Disorders**

| **Mental health condition** | **SNOMED CODE** | **ICD-9-cm**  **(ICD-10-cm) in DSM-5** |
| --- | --- | --- |
| Other depressive episodes | 268705006 | 311 (F32.8) or 311 (F32.9) |
| Recurrent depressive disorder, unspecified | 268710005 | 296.30 (F33.9) |
| Recurrent major depressive episodes | 268621008 | 296.33 (F33.2) |
| Recurrent major depressive episodes, in full remission | 191615005 | 296.36 (F33.42) |
| Depression | 35489007 | Not in DSM-5 |
| Depressive conduct disorder | 231542000 | Not in DSM-5 |
| Depressive episode, unspecified | 268706007 | 311 (F32.9) |
| Single major depressive episode, moderate | 191602001 | 296.22 (F32.1) |
| Dysthymia | 78667006 | 300.4 (F34.1) |
| Mixed anxiety and depressive disorder | 231504006 | Not in DSM-5 |
| Other recurrent depressive disorders | 192380004 | 296.30 (F33.9) |
| Mild depression | 310495003 | 296.21 (F32.0) |
| Mild recurrent major depression | 40379007 | 296.31 (F33.0) |
| Postpartum depression | 58703003 | Not in DSM-5 |
| Severe major depression, single episode, with psychotic features | 430852001 | 296.24 (F32.3) |
| Severe recurrent major depression with psychotic features | 28475009 | 296.34 (F33.3) |
| Severe recurrent major depression without psychotic features | 36474008 | 296.33 (F33.2) |
| Moderate recurrent major depression | 18818009 | - 1. (F33.1) |

**Additional table 4: Mental health conditions grouped under** **anxiety disorders**

| **Mental health condition** | **SNOMED CODE** | **ICD-9-cm**  **(ICD-10-cm) in DSM-5** |
| --- | --- | --- |
| Separation anxiety | 126943008 | 309.21 (F93.0) |
| Specific (isolated) phobias | 192396001 | 300.29 |
| Social phobia | 25501002 | 300.23 (F40.10) |
| Simple phobia | 54587008 | Not in DSM-5 |
| Agoraphobia | 70691001 | 300.22 (F40.00) |
| Agoraphobia with panic attacks | 191722009 | Not in DSM-5 |
| Agoraphobia without history of panic disorder | 61569007 | Not in DSM-5 |
| Panic disorder | 371631005 | 300.01 (F41.0) |
| Alcohol-induced anxiety disorder | 34938008 | In DSM-5 no ICD code |
| Anxiety disorder | 197480006 | Not in DSM-5 |
| Anxiety disorder, unspecified | 192405006 | 300.00 (F41.9) |
| Generalised anxiety disorder | 21897009 | 300.02 (F41.1) |
| Organic anxiety disorder | 17496003 | 293.84 (F06.4) |
| Other mixed anxiety disorders | 192403004 | Not in DSM-5 |
| Other phobic anxiety disorders | 192397005 | (F40.298) |
| Other specified anxiety disorders | 268714001 | 300.09 (F41.8) |
| Phobic anxiety disorder, unspecified | 192398000 | Not in DSM-5 |

**Additional table 5: Mental health conditions grouped under trauma and Stress related disorders**

| **Mental health condition** | **SNOMED CODE** | **ICD-9-cm**  **(ICD-10-cm) in DSM-5** |
| --- | --- | --- |
| Posttraumatic stress disorder | 47505003 | 309.81 (F43.10) |
| Acute stress disorder | 67195008 | 308.3 (F43.0) |
| Adjustment disorder | 17226007 | In DSM-5 no ICD code |
| Reactive attachment disorder of early childhood | 44124003 | Not in DSM-5 |

**Additional table 6: Mental health conditions grouped under** **personality disorders**

| **Mental health condition** | **SNOMED CODE** | **ICD-9-cm**  **(ICD-10-cm) in DSM-5** |
| --- | --- | --- |
| Cluster A personality disorder | 16805009 | In DSM-5 no ICD code |
| Paranoid personality disorder | 13601005 | 301.0 (F60.0) |
| Schizoid personality disorder | 52954000 | 301.20 (F60.1) |
| Cluster B personality disorder | 4306003 | In DSM-5 no ICD code |
| Antisocial personality disorder | 26665006 | 301.7 (F60.2) |
| Borderline personality disorder | 20010003 | 301.83 (F60.3) |
| Histrionic personality disorder | 55341008 | 301.50 (F60.4) |
| Narcissistic personality disorder | 80711002 | 301.81 (F60.81) |
| Cluster C personality disorder | 83890006 | In DSM-5 no ICD code |
| Avoidant personality disorder | 37746008 | 301.82 (F60.6) |
| Dependent personality disorder | 84466009 | 301.6 (F60.7) |
| Obsessive compulsive personality disorder | 1376001 | 301.4 (F60.5) |
| Other organic personality and behavioural disorders due to brain disease, damage, and dysfunction | 268680006 | 310.1 (F07.0) |
| Other specific personality disorders | 268728007 | 301.89 (F60.89) |
| Unspecified disorder of adult personality and behaviour | 192530006 | 301.9 (F60.9) |
| Mixed and other personality disorders | 192495001 | Not in DSM-5 |

**Additional table 7: Type of drug related dependence included in the drug dependence variable**

| Amphetamine dependence |
| --- |
| Benzodiazepine dependence |
| Buprenorphine-suboxone dependence-use (illicit use) |
| Cannabis dependence |
| Cannabis type drug dependence |
| Heroin dependence |
| Methadone dependence-use (illicit use) |
| Opioid dependence |
| Polysubstance dependence |
| Stimulant dependence |
| Gamma-hydroxybutyric acid (GHB) dependence |
| Psychostimulant dependence |
| Suboxone dependence |
| Cocaine dependence |
| Sedative dependence |
| Inhalant dependence |
| Hallucinogen dependence |
| Opium dependence |
